# Severe Malaria Infection Concurrent With Autoimmune Hemolytic Anemia and Acute Cytomegalovirus Infection: A Case Report

**DOI:** 10.7759/cureus.81597

**Published:** 2025-04-02

**Authors:** Shuaib Alsibai, Mohamad K Mohamad, Yousef S Alabrach, Ahmed Osman

**Affiliations:** 1 College of Medicine, Lincoln University College, Petaling Jaya, MYS; 2 Internal Medicine, Sheikh Khalifa Medical City, Abu Dhabi, ARE

**Keywords:** aiha, cmv, fever in traveler, malaria, sle

## Abstract

Malaria infection causes lysis of infected red blood cells (RBCs), leading to anemia. Autoimmune hemolytic anemia (AIHA), on the other hand, is caused by antibody-mediated destruction of RBCs, and it is associated with other autoimmune disorders like systemic lupus erythematosus (SLE). Thereby, we report a rare case of concurrent malaria infection with AIHA and acute cytomegalovirus (CMV) infection. A 33-year-old female presented with fever for seven days. She came back from Ethiopia 40 days ago. She was found to have severe anemia, and further workup revealed high levels of immunoglobulin G (IgG) antibodies against RBC. She rapidly improved with antimalarial and prednisolone therapy. She was also found to have immunoglobulin M (IgM) against CMV, indicating acute infection, but she didn't have specific manifestations related to CMV and therefore was not treated. During outpatient follow-up, she was found to have high antinuclear antibody (ANA) titers as well as low complements, indicating the possibility of an underlying autoimmune disorder. This case underscores the need for concurrent antimalarial and immunosuppressive therapy in co-occurring autoimmune hemolytic anemia (AIHA)/malaria, with close monitoring for SLE progression given high ANA titers.

## Introduction

Malaria-induced hemolysis is primarily due to the destruction of red blood cells (RBCs) parasitized by *Plasmodium *species, alongside immune-mediated lysis of uninfected RBCs [1]. In contrast, autoimmune hemolytic anemia (AIHA), which arises from autoantibodies against RBC antigens and is frequently associated with other autoimmune disorders [2]. Cytomegalovirus (CMV) infection is a possible trigger for AIHA as well [3]. The positive direct antiglobulin test (DAT) observed in AIHA may also appear in malaria, complicating its utility as a distinguishing marker [4]. Evidence suggests that malaria infection may trigger or exacerbate autoimmune conditions, including systemic lupus erythematosus (SLE) [5].

Clinical manifestations of both malaria and AIHA can overlap, as both can lead to manifestations of hemolytic anemia such as pallor, reduced hemoglobin count, and reduced haptoglobin [1,2]. However, fever, especially high-grade fever, is a more prominent feature of malaria [1]. Severe malaria can cause a picture of multisystem disease [1]. On the other hand, AIHA is associated with different etiologies, and therefore, the manifestations will depend on those disorders [6]. Moreover, AIHA can be the first manifestation of SLE [7]. Modern malaria treatment consists of artemisinin-based combination therapy, while treatment of AIHA relies on immunosuppression [1,2].

DAT positivity in both malaria and AIHA complicates diagnosis, necessitating parasite quantification, autoimmune serology, and response to therapy for differentiation. Thereby, we present a case of malaria vivax complicated by concurrent CMV infection and AIHA. The differentiation between the two entities poses a diagnostic challenge. This case also demonstrates the role of steroids in mixed hemolysis.

## Case presentation

A 33-year-old female not known to have any previous significant medical condition presented with bilateral lower limb swelling, fever, and chills for seven days prior to her presentation. She had traveled to Ethiopia 40 days prior to the presentation. She reported that none of her close contacts were having active symptoms, and she denied any bleeding episodes. Menstruation was normal with no heavy bleeding. Upon examination, the patient was febrile with a temperature of 40.5 °C with no specific pattern and tachycardic in the range of 100-150 beats per minute. She was otherwise vitally stable. Her skin was pale, and she had significant jaundice, but no hepatosplenomegaly was appreciated. She had significantly lower limb edema up to the thighs.

Blood tests were sent, and she was found to have hemolytic and macrocytic anemia, evident by hemoglobin of 57 (g/l), MCV of 121, high direct bilirubin of micromol/L units, and undetectable haptoglobin. She had thrombocytopenia with a platelet count of 89 x 10^9^/L (Table 1). She was admitted as a case of fever in a returning traveler with hemolytic anemia and thrombocytopenia. Therefore, diagnostic tests for infections and autoimmune disorders were sent. She was transfused with one RBC unit.

**Table 1:** Patient relevant positive lab findings Hg: hemoglobin; DAT: direct antiglobulin test; IgG: immunoglobulin G; IgM: immunoglobulin M; CMV: cytomegalovirus; ANA: antinuclear antibody

At presentation	During admission	At follow-up
Hg 57 g/L	Folate 5.8 nmol/L	Low C3 0.83 (normal range: 0.90-1.80)
Platelets 89 x 10^9^/L	Positive DAT Poly with +2 IgG	Low C4 0.10 (normal range: 0.10-0.40)
Direct bilirubin 15.7 micromol/L	Positive CMV IgM	ANA 1:640 (high titer)

Her peripheral blood smear was positive for *Plasmodium vivax, *and therefore, intravenous artesunate was initiated at a dose of 2.4 mg/kg, and scheduled for three doses; immediately, after 12 hours, and after 24 hours, given her severe picture. Moreover, folate level was low, so supplementation was given, and albumin level was 19 g/L, but she had no proteinuria. The hemolytic screen came positive for direct antiglobulin with +2 immunoglobulin G (IgG) and positive C3d. The hematology team was consulted, and they believe that her positive malaria screening might not explain her high autoimmune markers and severe derangement in her blood, and decided that the patient might benefit from a steroid course with a presumed diagnosis of AIHA. Therefore, the patient was started on prednisolone with a dose of 1 mg/kg.

Further testing revealed positive CMV immunoglobulin M (IgM) by chemiluminescence. However, the patient didn’t have organ-specific manifestations of CMV such as CMV pneumonitis or colitis. Therefore, as per the infectious disease team, there was no indication to treat at that time.

**Figure 1:**
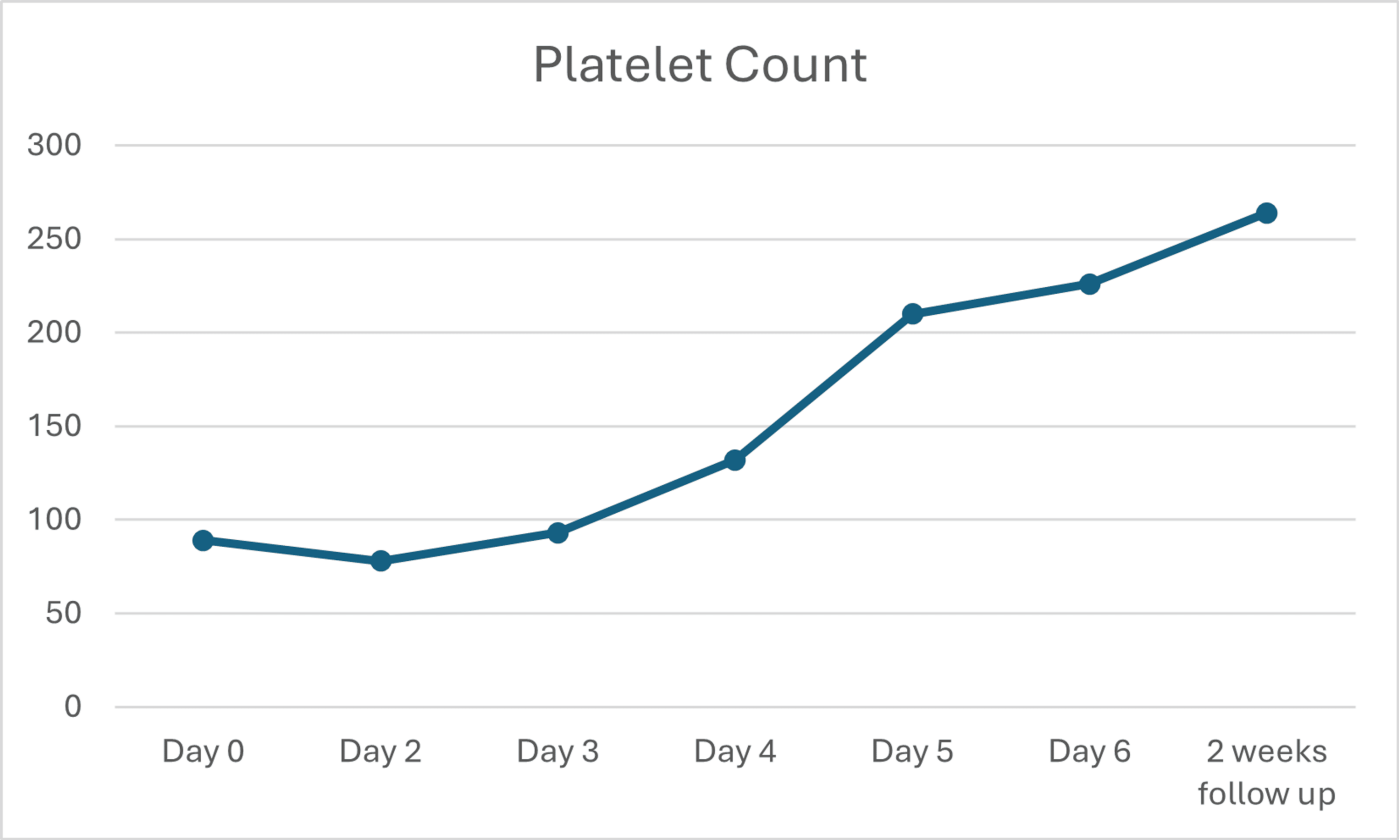
Improvement in the platelet count demonstrated over time

Patient clinically improved on artesunate and steroids, hemoglobin counts stabilized, and the platelets count improved (Figure 1). Patient was switched to the artemisinin-based oral regimen and then discharged on oral primaquine and a tapering prednisolone regimen; the patient was instructed to decrease the dose by 10 mg every week until stopped.

Following up in the clinic revealed a significant improvement in her hemoglobin count. Antinuclear antibody (ANA) came out after discharge to be positive with a titer of 640, and her C3 and C4 counts were low, but double-stranded DNA (DsDNA) was negative (Table 1). The patient was doing well on further checkups with no evidence of developing SLE.

## Discussion

This case presents a complex interplay between AIHA and *P. vivax *malaria, both of which are associated with hemolysis. Given the positive ANA and low complements, this further strengthens the possibility of having concurrent AIHA with her malaria [8]. Moreover, especially given her demographics, it could represent an early presentation of SLE.

**Figure 2:**
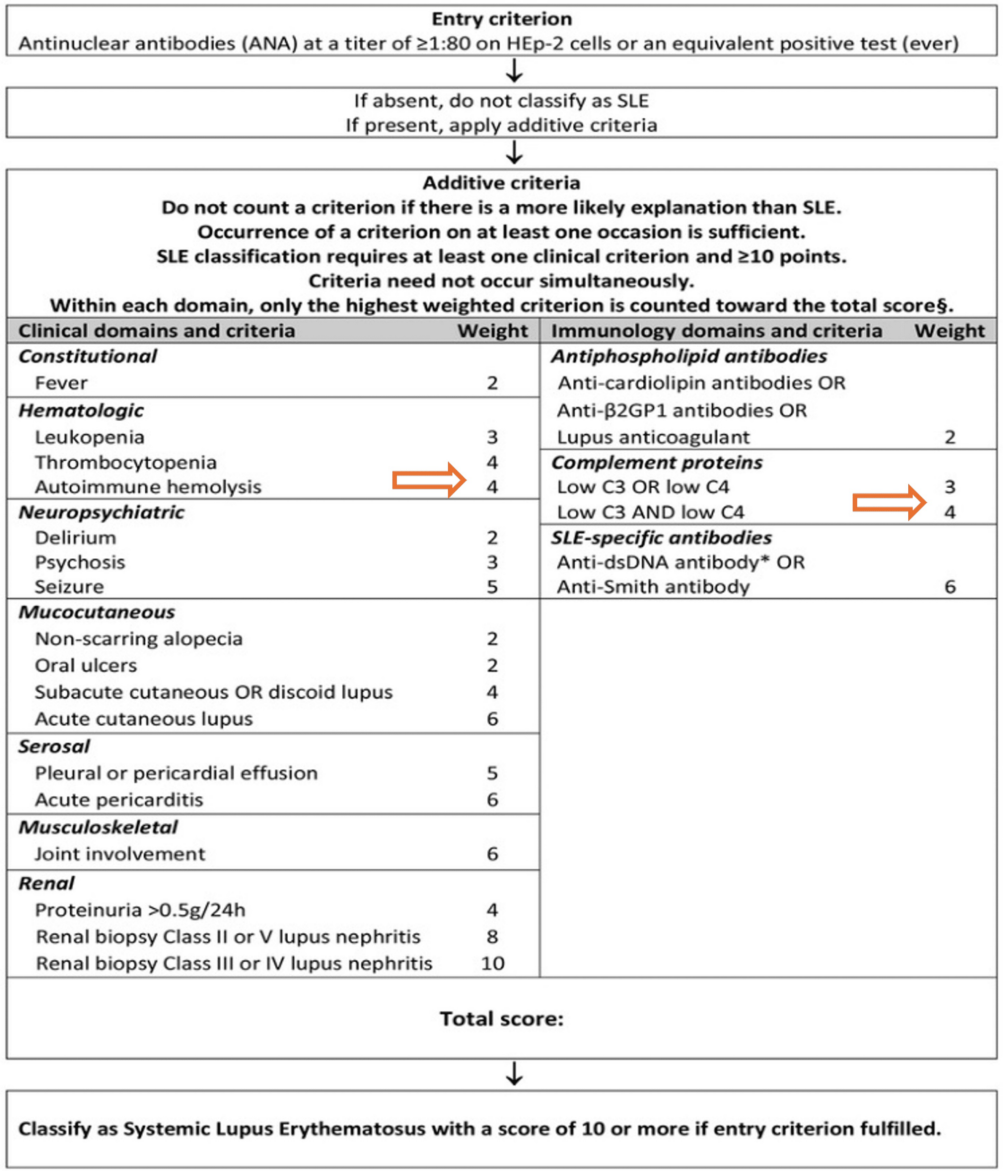
SLE EULAR/ACR 2019 criteria. Patient should have a score of 10; our patient score is 8 after excluding fever since she has malaria. SLE: systemic lupus erythematosus; EULAR/ACR: European League Against Rheumatism/American College of Rheumatology Credit: Adapted from [9]

Although the patient doesn’t fulfill the criteria of SLE, given the lack of other features and the absence of SLE-specific antibodies (Figure 2) [9], this might be an early presentation, especially given the language barrier and lack of in-depth rheumatological history. What supports the presence of long-standing autoimmune disease and hemolysis is the folate level, which is not expected to happen acutely [10].

Interestingly, genetic factors associated with SLE may modulate susceptibility and response to malaria. Studies have demonstrated that SLE-associated polymorphisms, such as those in the FcγRIIB receptor, enhance phagocytosis of malaria-infected RBCs, leading to increased parasite clearance and potentially reduced disease severity [11,12]. This genetic advantage, likely an evolutionary adaptation in malaria-endemic regions, also predisposes individuals to autoimmune disorders like SLE [12]. Genetic testing was not performed in this case but can be considered in future visits. Moreover, it is well known that pre-existing autoimmune disorders increase susceptibility to infection as well as the severity of it. This might explain her severe picture, given that anemia caused by *P. vivax* is usually less severe than *Plasmodium* *falciparum *[13,14]. Only one previously reported case showed the concurrent presence of *P. vivax* and direct antiglobulin reactions [14].

CMV is also a rare trigger for AIHA [3]. CMV in an immunocompetent host usually presents with mononucleosis-like illness, which is characterized by fever, rash, and leukocytosis [15]. The patient didn't have rash, and her labs revealed leukopenia. Moreover, organ-specific involvement was not present in this case since the patient is not severely immunocompromised. What it could have contributed to is the fever and also the positive ANA titers [15]. Moreover, it could have served as a trigger for AIHA or even SLE, as this relation is reported before [16]. Measuring CMV viral load might have assisted in decision-making, but unfortunately, it was not performed. Nevertheless, the treatment would most likely remain supportive since there is no organ-specific involvement. The patient's rapid clinical improvement without CMV-directed therapy might indicate that CMV was not a significant contributor to her presentation.

Conversely, the immunosuppressive treatments used in AIHA and/or SLE, such as corticosteroids and cyclophosphamide, can exacerbate infections like malaria [17]. This interplay complicates treatment strategies, as the management of malaria-induced hemolysis (antimalarial therapy) and AIHA (immunosuppression) may conflict. In this patient, the dual approach of antimalarial therapy with artesunate and immune modulation with corticosteroids was essential to control both conditions.

## Conclusions

This case highlights malaria-AIHA interplay, with SLE potential. It emphasizes the intricate relationship between autoimmunity and infectious diseases. It also underscores the need for a thorough diagnostic workup to identify the etiology of hemolysis in patients with concurrent AIHA/SLE and malaria. Laboratory investigations, including peripheral blood smear, parasitemia levels, and DAT results, alongside clinical response to therapy, are critical. Moreover, future SLE monitoring is important to aid in the early detection of progression to SLE. CMV infection might have also contributed to her clinical presentation. Further research into the genetic and immunological factors underlying this interplay is necessary to optimize diagnostic and therapeutic approaches.

## References

[1] White NJ (2018). Anaemia and malaria. Malar J.

[2] Go RS, Winters JL, Kay NE (2017). How I treat autoimmune hemolytic anemia. Blood.

[3] Romano S, Pepe G, Fotzi I, Casini T, Chiocca E, Trapani S (2023). Autoimmune hemolytic anemia (AIHA) secondary to cytomegalovirus (CMV) infection in a 2-month-old infant: a case report. Children (Basel).

[4] Camprubí D, Pereira A, Rodriguez-Valero N (2019). Positive direct antiglobulin test in post-artesunate delayed haemolysis: more than a coincidence?. Malar J.

[5] Bansal R, Yadav A, Raizada A, Sharma S, Goel A (2017). Can malaria trigger systemic lupus erythematosus?. Trop Doct.

[6] Michalak SS, Olewicz-Gawlik A, Rupa-Matysek J, Wolny-Rokicka E, Nowakowska E, Gil L (2020). Autoimmune hemolytic anemia: current knowledge and perspectives. Immun Ageing.

[7] Kokori SIG, Ioannidis JPA, Voulgarelis M, Tzioufas AG, Moutsopoulos HM (2000). Autoimmune hemolytic anemia in patients with systemic lupus erythematosus. Am J Med.

[8] Shin SY, Yu JH, Kim JY, Kim YJ, Woo HY, Kwon MJ, Yeom JS (2012). A case of mixed malaria infection with severe hemolytic anemia after travel to Angola. Infect Chemother.

[9] Aringer M, Costenbader K, Daikh D (2019). 2019 European League Against Rheumatism/American College of Rheumatology classification criteria for systemic lupus erythematosus. Ann Rheum Dis.

[10] Khan KM, Jialal I (2023). Folic acid deficiency. StatPearls [Internet].

[11] Clatworthy MR, Willcocks L, Urban B (2007). Systemic lupus erythematosus-associated defects in the inhibitory receptor FcgammaRIIb reduce susceptibility to malaria. Proc Natl Acad Sci U S A.

[12] Waisberg M, Tarasenko T, Vickers BK (2011). Genetic susceptibility to systemic lupus erythematosus protects against cerebral malaria in mice. Proc Natl Acad Sci U S A.

[13] Geleta G, Ketema T (2016). Severe malaria associated with Plasmodium falciparum and P. vivax among children in Pawe hospital, northwest Ethiopia. Malar Res Treat.

[14] Lee SW, Lee SE, Chung BH, Hwang TJ, Shin HS (2008). A case of Plasmodium vivax malaria associated with autoimmune hemolytic anemia. Infect Chemother.

[15] (2025). Cytomegalovirus - StatPearls - NCBI Bookshelf. https://www.ncbi.nlm.nih.gov/books/NBK459185/.

[16] Yamazaki S, Endo A, Iso T (2015). Cytomegalovirus as a potential trigger for systemic lupus erythematosus: a case report. BMC Res Notes.

[17] He J, Li Z (2023). Dilemma of immunosuppression and infection risk in systemic lupus erythematosus. Rheumatology (Oxford).

